# Overexpression of *Rice Black-Streaked Dwarf Virus* P7-1 in *Arabidopsis* Results in Male Sterility Due to Non-Dehiscent Anthers

**DOI:** 10.1371/journal.pone.0079514

**Published:** 2013-11-15

**Authors:** Feng Sun, Xia Yuan, Qiufang Xu, Tong Zhou, Yongjian Fan, Yijun Zhou

**Affiliations:** Key Laboratory of Monitoring and Management of Plant Virus Diseases, Institute of Plant Protection, Jiangsu Academy of Agricultural Sciences, Nanjing, Jiangsu Province, China; Nanjing Agricultural University, China

## Abstract

Rice black-streaked dwarf virus (RBSDV), a member of the genus *Fijivirus* in the family *Reoviridae*, is propagatively transmitted by the small brown planthopper (*Laodelphax striatellus* Fallén). RBSDV causes rice black-streaked dwarf and maize rough dwarf diseases, which lead to severe yield losses in crops in China. Although several RBSDV proteins have been studied in detail, the functions of the nonstructural protein P7-1 are still largely unknown. To investigate the role of the P7-1 protein in virus pathogenicity, transgenic *Arabidopsis thaliana* plants were generated in which the P7-1 gene was expressed under the control of the 35S promoter. The RBSDV P7-1-transgenic *Arabidopsis* plants (named P7-1-OE) were male sterility. Flowers and pollen from P7-1-transgenic plants were of normal size and shape, and anthers developed to the normal size but failed to dehisce. The non-dehiscent anthers observed in P7-1-OE were attributed to decreased lignin content in the anthers. Furthermore, the reactive oxygen species levels were quite low in the transgenic plants compared with the wild type. These results indicate that ectopic expression of the RBSDV P7-1 protein in *A. thaliana* causes male sterility, possibly through the disruption of the lignin biosynthesis and H_2_O_2_-dependent polymerization pathways.

## Introduction

Rice black-streaked dwarf virus (RBSDV), a member of the genus Fijivirus in the family *Reoviridae*, causes rice black-streaked dwarf and maize rough dwarf diseases, which lead to severe yield losses in crops in China, Japan, Korea, and other Asian countries [1], [2], [3]. The virus is propagatively transmitted by the small brown planthopper (SBPH: *Laodelphax striatellus* Fallén) in a persistent circulative manner [1], [3]–[5]. Plants infected with RBSDV typically exhibit symptoms such as stunting, darkening of leaves, and white waxy or black-streaked swellings along the veins on the backs of the leaf blades and sheaths [1], [3]–[5].

The RBSDV virion is composed of icosahedral, two-layered particles approximately 75–80 nm in diameter and 10 segments of double-stranded genomic RNA (dsRNA), which are designed S1 to S10 with increasing order of electrophoretic mobility in polyacrylamide gel electrophoresis (PAGE) [6], [7]. S1 encodes a putative 169-kDa RNA-dependent RNA polymerase, while S2 and S4 encode a major core structural protein and the outer shell B-spike protein, respectively [6], [7], [8]. The protein encoded by S3 contains a conserved motif and is a putative guanylyltransferase [9]. Western blotting analysis of viral particles has suggested that the minor core capsid protein and major outer capsid protein are encoded by S8 and S10, respectively [8], [10], [11]. Immunoelectron microscopy has revealed that S9 ORF1 P9-1 is a nonstructural protein that accumulates in the intracellular viroplasms in infected plants and insects [8]. P9-1, a thermostable, α-helical protein, self-interacts to form dimers and is the minimal viral component required for viroplasm formation [12]. The protein encoded by S6 is a viral RNA silencing suppressor and has an intrinsic ability to interact with P9-1 [13]–[15].

P7-1 is a nonstructural protein containing 363 amino acids (with a molecular mass of 41.0 kDa) that is translated from S7 ORF1. P7-1 accumulates in tubule-like structures in infected plant and planthopper cells, suggesting that P7-1 is one of the constituents of these structures [8]. Recent studies demonstrate that RBSDV P7-1 forms punctuate points at plasmodesmata in *Nicotiana benthamiana* leaves [16]. Tubule-forming and plasmodesmatal localization of RBSDV P7-1 are usually thought to be involved in cell-to-cell movement of the virus in plant and insect cells. However, little else is known about the characteristics and functions of P7-1. In this study, we determined that RBSDV P7-1 is a pathogenicity determinant and that this protein affects normal plant development when expressed as transgenes in *Arabidopsis*. RBSDV P7-1 transgenic *Arabidopsis* plants exhibited male sterility phenotype caused by a defect of secondary wall lignification in the anther endothecium and failure of anther dehiscence. Furthermore, non-dehiscent anthers observed in transgenic plants were accompanied by decreased levels reactive oxygen species (ROS). These results suggest that impairment in lignin biosynthesis and the H_2_O_2_-dependent polymerization in RBSDV P7-1 over-expressing *Arabidopsis* plants cause male sterility.

## Materials and Methods

### Virus Isolates, Vectors, and Plant Materials

Rice plants infected with RBSDV were collected from Jiangsu Province in China under the permission of Plant Protection Station of Jiangsu Province, China. Young instar nymphs of SBPHs (small brown planthopper, *L.striatellus*) were fed RBSDV-infected rice plants for 2 days to acquire the virus. A dot immunobinding assay (DIBA) [3] showed that 20–30% of adult insects were viruliferous. A virus-free planthopper population was generated using a pair of recently hatched nymphs, and nonvirulence was confirmed using reverse transcription-polymerase chain reaction (RT-PCR). Viruliferous or virus-free SBPHs were reared on healthy rice seedlings (*Oryza sativa* L. japonica. cv. Wuyujing No. 3) in glass vessels at 22°C under alternating photoperiods of 14 h of light and 10 h of dark.

*Arabidopsis* (Col-0) seeds were donated by Prof. Hansong Dong (College of Plant Protection, Nanjing Agricultural University, Nanjing, China). Plants were grown in potting soil in a growth chamber at 24°C under 200 µE·m^−2^·s^−1^ illumination and 16-h light/8-h dark cycle conditions.

### RBSDV Inoculation Assay

Rice plants (*Oryza sativa* L. japonica. cv. Nipponbare) were inoculated with 10 viruliferous (RBSDV) or virus-free (mock) nymphs per plant (4 or 5 leaves) and were kept in a growth chamber containing 30 plants. After incubation at 22°C for 4 days under artificial light, planthoppers were removed. Plants were maintained in field conditions for symptom development.

### Construction of Overexpression Vector and *Arabidopsis* Transformation

To generate the RBSDV P7-1 overexpression plasmid, the coding region of the RBSDV P7-1 gene (from +1 to +1,089 bp) was amplified with primers containing the *Sac*I and *Bam*HI sites (Table S1). The resulting PCR product was digested with *Sac*I and *Bam*HI and ligated into the *Sac*I-*Bam*HI-cleaved pCHF3 vector. After DNA sequencing, the RBSDV P7-1 overexpression plasmid was introduced into *Agrobacterium tumefaciens* (strain EHA105), and the transformation of *Arabidopsis thaliana* Col-0 ecotype was performed by the floral-dip method [17]. Homozygous T3 generation plants were screened for kanamycin resistance and identified with genome PCR. RBSDV P7-1 gene sequence has been deposited in NCBI Genbank (http://www.ncbi.nlm.nih.gov) under the accession number of KF532967.

### Qenome DNA PCR

Arabidopsis genome DNA was isolated from leaves using SQ Plant DNA Kit (Omega Bioservices; Norcross, GA, USA) according to the manufacturer’s instructions. PCR reaction was performed using 10 µl Premix Taq (TAKARA; Dalian, China), 0.2 µM RBSDV S7-1 specific forward and reverse primers (Table S1), and 1 µl DNA in a total volume of 20 µl. All samples were subjected to denaturation for 5 min at 95°C, followed by 30 cycles of 95°C for 30 s, 58°C for 30 s and 72°C for 1 min. PCR products were detected using agarose gel electrophoresis, and images were obtained using the Bio-Rad Molecular Imager Gel Doc XR System (Bio-Rad; Hercules; CA, USA) after staining with ethidium bromide.

### Quantitative Real-time PCR

Quantitative real-time PCR (qRT-PCR) was performed using SsoFast EvaGreen Supermix (Bio-Rad; Hercules; CA, USA) in a Bio-Rad iQ5 Real-Time PCR system. Total RNA was isolated from leaves using the RNAiso Plus reagent (TAKARA; Dalian, China) and reverse transcribed using M-MLV Reverse Transcriptase (Promega; Fitchburg; WI, USA), according to the manufacturer’s instructions. The *EF1α* gene, which is highly conserved and constitutively expressed in eukaryotes [18], was used as a reference control. Each quantitative PCR reaction was performed using 10 µl SsoFast EvaGreen Supermix, 0.2 µM forward and reverse primers, and 1 µl cDNA in a total volume of 20 µl. All samples were subjected to denaturation for 3 min at 95°C, followed by 40 cycles of 95°C for 10 s and 58°C for 20 s. SYBR Green absorbance was detected at 58°C. All reactions were conducted in triplicate. Amplicon dissociation curves, i.e., melting curves, were recorded after cycle 40 by heating from 60°C to 95°C at a ramp speed of 1.9°C·min^−1^. Data were analyzed using iQ5 software (Bio-Rad; Hercules; CA, USA). Table S1 shows information regarding additional genes and primers employed in this study.

### Observation of Lignified Secondary Wall Thickening

For ethidium bromide staining, fresh anthers were washed in 10 mM PBS and 2% (v/v) Tween20 for 10 min and 10 mM PBS for 10 min, followed by staining with 0.05% (w/v) ethidium bromide (1 h, room temperature). The anthers were then washed (10 mM PBS, 10 min), and the stained tissues were mounted on a glass slide and examined using Zeiss LSM710 confocal microscopy (Carl Zeiss Microscopy GmbH; Jena, Germany) under red fluorescence at 543 nm excitation [19].

To study lignin autofluorescence, the anthers were cleared in 70% (v/v) lactic acid for 3 days at 60°C, and the cleared tissues were examined using Zeiss LSM710 confocal microscopy (Carl Zeiss Microscopy GmbH; Jena, Germany) under UV light excitation.

For phloroglucinol–HCl staining, flowers were fixed in FAA solution overnight and decolorized with an ethanol series. The flowers were then stained with 2% (w/v) phloroglucinol in 92% ethanol for 1 h at room temperature. Subsequently, the tissues were mounted with 18.5% (v/v) HCl, and red staining was immediately monitored using Leica M125 microscopy (Leica Microsystems; Bannockburn; IL, USA).

### Histochemical Detection of ROS, H_2_O_2_, and O_2_^−^ Radical

The accumulation of ROS was visualized by a fluoluminescence assay with 2,7-dichlorofluorescein diacetate (DCFH-DA), which emits green fluorescence when oxidized by ROS. In this assay, anthers were infiltrated with a 100 µM/ml aquatic solution of DCFH-DA (Sigma; St. Louis; MO, USA) and subsequently observed with Zeiss LSM710 confocal microscopy (Carl Zeiss Microscopy GmbH; Jena, Germany) under GFP fluorescent light.

The H_2_O_2_ and O_2_^−^ levels were measured by 3, 3′-diaminobenzidine (DAB) and nitroblue tetrazolium (NBT) (Sigma; St. Louis; MO, USA) staining, respectively [20]. Anthers were infiltrated in DAB solution (0.1% w/v, pH 3.8) and incubated overnight in darkness at 22°C. Alternatively, the anthers were infiltrated in NBT solution (0.1% w/v) in 10 mM PBS (pH 7.8) containing 10 mM NaN_3_ and then incubated in darkness at 22°C for 1 h. After incubation, the stained tissues were decolorized with acetic acid: glycerol: ethanol (1∶1∶3, v/v/v) solution at 95°C for 10 min and then photographed using Leica M125 microscopy (Leica Microsystems; Bannockburn; IL, USA).

### Scanning Electron Microscopy

Tissue samples of wild-type and P7-1-OE flower bud clusters were fixed overnight in 50 mM phosphate-buffered saline (PBS, pH 6.8) containing 1% (v/v) glutaraldehyde and 2% (v/v) paraformaldehyde at 4°C. The fixed tissues were rinsed twice (15 min each) with 100 mM PBS at RT. The tissues were dehydrated in a graded ethanol series. Then, the samples were coated with gold and examined in an S-3000N Scanning Electron Microscope (Hitachi; Tokyo, Japan) at an accelerating voltage of 10 kV.

## Results

### Reproductive Development Symptoms Caused by RBSDV Infection in Rice Plants

**Figure 1:**
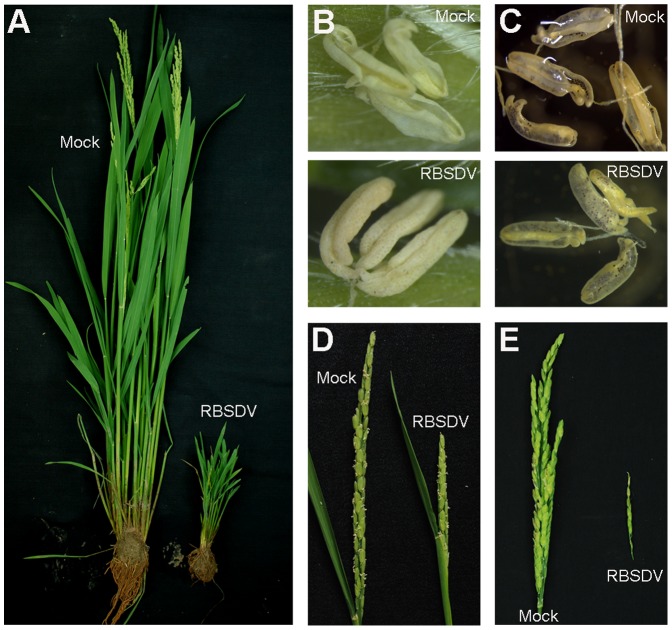
Symptoms of rice plants infected with *Rice black-streaked dwarf virus* (RBSDV). (A) The stunted symptom of rice caused by RBSDV infection. (B–C) RBSDV-infected rice plants showing non-dehiscent anthers (B) but normal viable pollen stained with I_2_ (C). (D–E) The spikelets of RBSDV-infected rice plants at flowering (D) and at the grain filling period (E). Rice plants were inoculated with viruliferous (RBSDV) or virus-free SBPHs (mock).

The growth and development of rice plants were affected by RBSDV infection. The typical symptoms of RBSDV-infected rice plants include severe stunting and darkening of leaves [3]–[5]. However, reproductive development in rice plants infected by RBSDV has not previously been studied in detail. Here, we characterized the reproductive development phenotype of RBSDV-infected rice plants, which were inoculated by viruliferous planthoppers, and we allowed disease symptoms to develop under field conditions. Two month after inoculation, the infected rice plants showed significantly stunted growth (Figure 1A) and produced poor spikelets (Figure 1D). At flowering stage, the spikelets of infected rice plants had viable pollen grains (Figure 1C), but the anthers failed to dehisce (Figure 1B) compared with mock rice plants which were inoculated by virus-free planthoppers. As a result, at grain filling stage the infected rice plant showed partial to complete spikelet sterility (Figure 1E).

### Expression of RBSDV P7-1 Protein Cause Male Sterility due to Non-dehiscent Anthers in *Arabidopsis*

**Figure 2:**
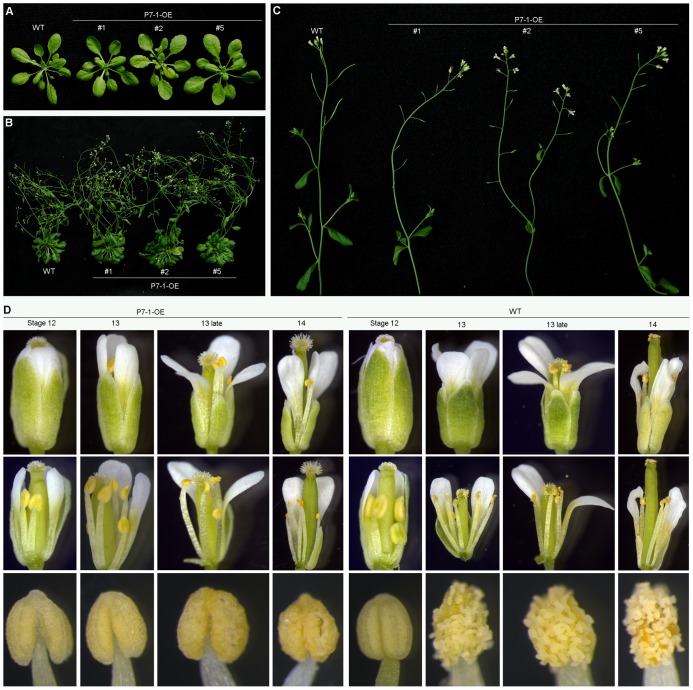
Ectopic expression of RBSDV P7-1 causes male sterility in *Arabidopsis*. (A) Phenotypes of P7-1 transgenic plants (P7-1-OE) and wild-type (WT) *Arabidopsis* during vegetative growth. (B) Phenotypes of P7-1 transgenic lines (P7-1-OE) and wild-type (WT) *Arabidopsis* during reproductive development. (C) Silique phenotypes of P7-1 transgenic lines (P7-1-OE) and wild-type (WT) *Arabidopsis*. (D) Developmental series of P7-1-OE and wild-type flowers during anther stages 12 to 14.

To determine whether proteins encoded by RBSDV contributed to the variation in anther dehiscence and spikelet fertility of virus-infected rice plants, we investigated the effects of P7-1 on *Arabidopsis* plants. Specifically, we expressed the full-length P7-1 cDNA, under the control of the 35S-CaMV promoter, in transgenic *Arabidopsis* plants. The transgenic plants that constitutively expressed RBSDV P7-1 were selected using kanamycin medium (Figure S1A) and confirmed by genomic PCR (Figure S1B) and quantitative real-time RT-PCR analysis (Figure S1C). T3 generation plants from transgenic lines exhibiting high levels of overexpression of P7-1 (P7-1-OE#1, #2, and #5) were selected for further study. During the vegetative growth stage, the growth and development of P7-1-OE#1, #2, #5, and wild-type *Arabidopsis* seedlings were indistinguishable (Figure 2A). However, all transgenic lines (P7-1-OE#1, #2, and #5) showed significant abnormalities during reproductive growth. Following fertilization, siliques of wild-type plants elongated and set seeds. But in transgenic plants, siliques remained small, producing no seeds, indicating the high degree of sterility of Arabidopsis plants ectopically expressing RBSDV P7-1 (Figure 2B and 2C).

**Figure 3:**
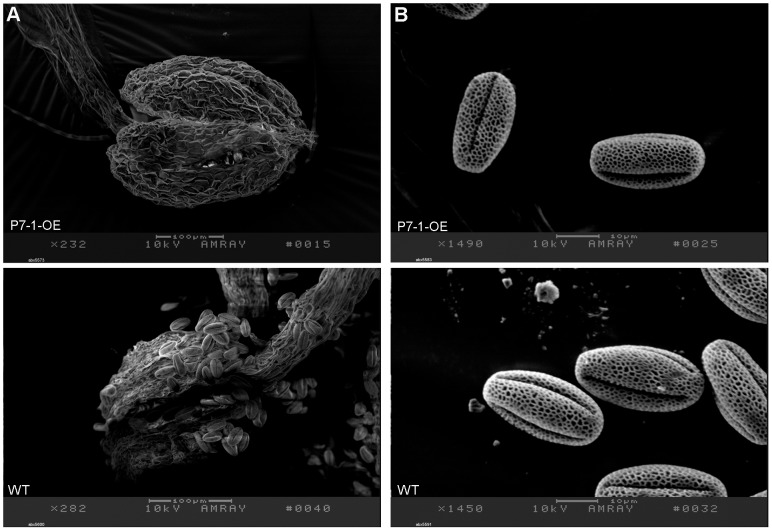
Scanning electron micrographs of anthers (A) and pollen grains (B) of P7-1 transgenic and wild-type (WT) *Arabidopsis*.

To investigate the cause of the sterile phenotype in transgenic plants overexpressing RBSDV P7-1, we examined the flowers under a microscope. These observations revealed that in wild-type flowers, the anthers started to dehisce at stage 13 [21], and pollen grains were released to the stigmatic papilla (Figure 2D). However, at stage 13, P7-1-OE transgenic anthers did not dehisce normally, resulting in the failure to release pollen grains to the stigmatic papilla (Figure 2D). Even at late stage 14, P7-1-OE anthers remained closed, and no pollen grains were released (Figure 2D, Figure S2A). Analysis of anther morphology by scanning electron microscopy showed that at late stage 13, wild-type anthers spilt open along the stomium and released pollen grains, whereas P7-1-OE anthers remained intact, and no pollen grains were observed (Figure 3A). Scanning electron microscopy and Alexander staining revealed that the pollen grains within the indehisced anthers of P7-1-OE plants were morphologically normal (Figure 3B) and viable (Figure S2B and S2C). In addition, when used for hand pollination, the P7-1-OE pollen grains could fertilize egg cells (Figure S3). These observations indicate that the sterility of the P7-1-OE lines is caused solely by defective anther dehiscence.

### P7-1-OE Anthers have Reduced Endothecium Secondary Wall Lignification

**Figure 4:**
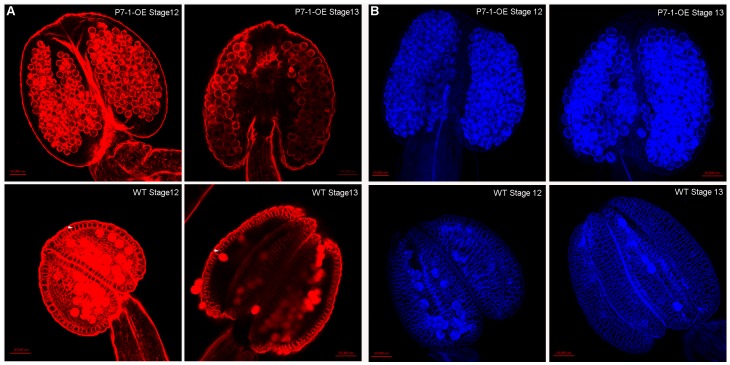
Endothecial secondary wall thickening in anthers from P7-1 transgenic (P7-1-OE) and wild-type plants. (A) Anthers were stained with ethidium bromide and visualized by confocal microscopy. Secondary thickening occurs in the endothecium (indicated by an arrow). Thickening is clearly observed in WT but is reduced P7-1 transgenic (P7-1-OE) endothecium. (B) Lactic acid-cleared anthers visualized by confocal microscopy. The secondary thickening network is clearly observed in WT plants, but not in P7-1 transgenic plants (P7-1-OE).

**Figure 5:**
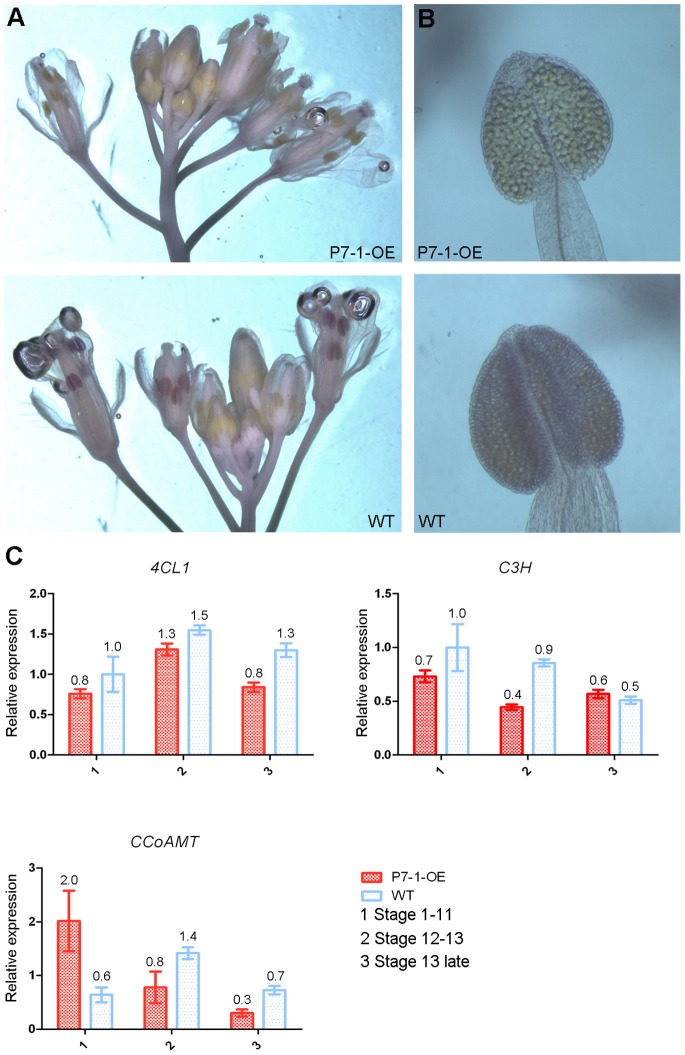
Histochemical staining of lignin components in P7-1 transgenic and WT *Arabidopsis*. (A) Phloroglucinol staining of P7-1 transgenic (P7-1-OE) and wild-type (WT) *Arabidopsis* flowers. Phloroglucinol-stained material was highly accumulated in WT anthers but not in P7-1-OE anthers during flower development. (B) Magnified views of P7-1-OE and WT anthers stained with phloroglucinol. (C) The expression levels of *4CL* (*4-coumarate:CoAligase*), *CCoAOMT* (*caffeoyl CoA O-methyltransferase*), and *C3H* (*cinnamic acid 3-hydroxylase*) were detected in P7-1 transgenic (P7-1-OE) and wild type (WT) *Arabidopsis* flowers using quantitative real-time polymerase chain reaction (qRT-PCR) with the *EF1a* gene as an internal standard. Results were presented as means±SE from three replications.

Secondary wall lignification, which mainly occurs around the endothecial cells, is thought to be critical for generating the forces required for anther dehiscence [22]. In wild-type plants, when the anthers were stained with ethidium bromide and visualized by confocal microscopy, lignified secondary walls could be observed in the endothecium as bands of striated spring-like thickenings at stages 12 and 13 (Figure 4A bottom). By contrast, this thickening was absent in the P7-1-OE plants (Figure 4A top). To confirm the anther wall structure, lactic acid was used to clear the anthers. Under UV-illumination, a net-like structure of autofluorescent material could be seen at stage 12 or 13 in wild-type plants, indicating that there was lignification of the anther wall (Figure 4B, bottom). However, these structures were not found in the P7-1-OE plants (Figure 4B, top). Furthermore, we investigated the lignification patterns in both wild-type and P7-1-OE anthers using phloroglucinol staining, which is a histochemical stain commonly used for specifically staining lignin [23]. Phloroglucinol-stained substances accumulated to a high degree in wild-type anthers during their development, both at stage 13 and after flowering (Figure 5A and 5B). By contrast, phloroglucinol staining was not observed in P7-1-OE anthers during flower development (Figure 5A and 5B). Moreover, the expression levels of the lignin biosynthesis genes *4CL* (*4-coumarate:CoAligase*), *CCoAOMT* (*caffeoyl CoA O-methyltransferase*), and *C3H* (*cinnamic acid 3-hydroxylase*) were low in P7-1-OE flowers, especially at stage 13 or later, compared with wild-type flowers (Figure 5C). For example, at flowers development 13 stage, the relative expression level of *4CL*, *CCoAOMT* were 0.8, 0.3 in P7-1-OE plants but 1.3, 0.7 in wild-type plants.

### Anthers of P7-1-OE Plants have Reduced ROS Level

**Figure 6:**
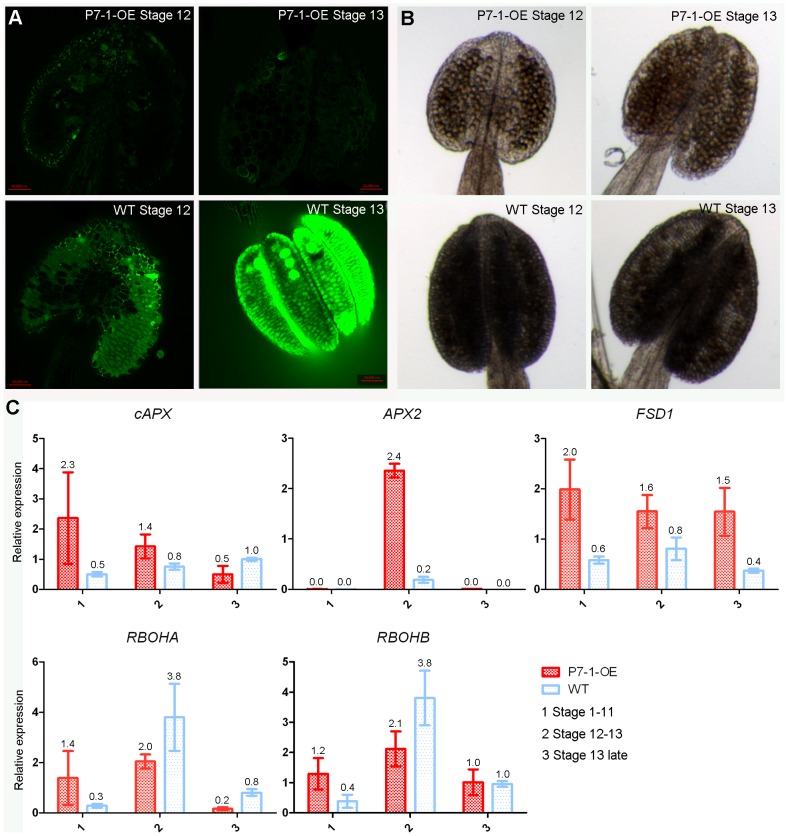
Anthers from P7-1 transgenic plants exhibit low ROS and H_2_O_2_ contents compared with WT plants. (A) ROS was detected by staining with DCFH-DA and visualized by confocal microscopy. (B) H_2_O_2_ was detected by staining with DAB. (C) The expression levels of *cytosolic ascorbate peroxidase1*(*cAPX1*), *ascorbate peroxidase2* (*APX2*), *Fe superoxide dismutase* (*FSD1*), *respiratory burst oxidase homolog A* (*RBOHA*), and *respiratory burst oxidase homolog B* (*RBOHB*) were detected in P7-1 transgenic (P7-1-OE) and wild type (WT) *Arabidopsis* flowers using qRT-PCR with the *EF1a* gene as an internal standard. Results were presented as means±SE from three replications.

To assess whether the sterility of P7-1-OE flowers was related to plant signaling molecules, we measured the ROS and NO (nitric oxide) levels in both transgenic and wild-type anthers using florescence staining. Our results clearly demonstrated that there was no difference in NO content between P7-1-OE and wild-type anthers (Figure S4). However, the transgenic anthers accumulated significantly lower levels of ROS than wild-type plants (Figure 6A). We further investigated ROS production in anthers during development by histochemical staining. P7-1-OE anthers produced low levels of H_2_O_2_ (Figure 6B, DAB staining) but equivalent amounts of O_2_^−^ (Figure S5, NBT staining) compared with wild-type plants. To gain further insight into the low ROS levels in P7-1-OE anthers, we analyzed the expression levels of genes in the ROS network. The expression levels of some genes that encode ROS-scavenging enzymes, e.g., *cytosolic ascorbate peroxidase1* (*cAPX1*), *ascorbate peroxidase2* (*APX2*), and *Fe superoxide dismutase* (*FSD1*), increased in the P7-1-OE anthers about 2-fold higher than in wild-type, but the levels of ROS-producing related genes, e.g., *respiratory burst oxidase homolog A* (*RBOHA*) and *respiratory burst oxidase homolog B* (*RBOHB*), were reduced in the transgenic anthers compared with the wild type (Figure 6C). These results show that the accumulation of ROS in anthers prior to dehiscence is nearly absent when RBSDV P7-1 is ectopically expressed.

## Discussion

Rice black-streaked dwarf and maize rough dwarf diseases, which are caused by RBSDV, lead to severe yield losses in crops in southeast Asian countries, especially China [3], [4]. Although a great deal of effort has been made to elucidate the interactions between the virus, insect vectors, and host and environmental conditions, few RBSDV proteins involved in pathogenesis have been identified, and the biochemical basis of disease symptom development remains largely unknown. In this study, our results suggest that RBSDV P7-1 is a novel determinant of disease symptom development. First, RBSDV infection caused alterations in anther development in rice plants, resulting in partial sterility due to non-dehiscent anthers (Figure 1). Furthermore, overexpressing RBSDV P7-1 in *Arabidopsis* produced plants with male sterility resulting from non-dehiscent anthers (Figure 2), which approximately mimicked the disease symptoms observed in rice anthers. The non-dehiscent anther phenotype of P7-1 transgenic plants was correlated with reduced secondary wall lignification in the endothecium and decreased ROS levels in the anthers (Figure 5 and 6). Taken together, these results suggest that RBSDV P7-1 is likely to be a pivotal determinant of plant sterility symptoms caused by RBSDV.

Previous studies using immunoelectron analysis have revealed that RBSDV P7-1 accumulates in tubular structures in virus-infected maize plants and in insects [8]. Furthermore, P7-1 of SRBSDV (southern rice black-streaked dwarf virus), which is most closely related to RBSDV, has the intrinsic ability to form tubules growing from the non-host insect cell surface in the absence of other virus proteins [24]. Recent researches using live-cell imaging in *Nicotiana benthamiana* leaves also demonstrate that RBSDV P7-1 protein forms punctuate points at plasmodesmata [16]. RBSDV P7-1 characteristics of tubule-forming and plasmodesmata localization suggest that this protein might be involved in virus intercellular movement, in infected insect and plant cells. The pathogenesis of tubular proteins during viral morphogenesis has been reported in studies of other members of the family *Reoviridae.* For example, BTV (bluetongue virus) nonstructural tubular protein NS1 plays a direct role in cellular pathogenesis [25]. RDV (rice dwarf virus) nonstructural tubular protein Pns10 functions as a viral suppressor of RNA silencing and plays important roles in enhancing viral replication, systemic movement, and invasion of new tissues [26]. Therefore, the tubular proteins of reoviruses have multiple functions in viral morphogenesis, viral infection, and virulence. In support of this notion, our data show that overexpression of RBSDV tubular protein P7-1 in *Arabidopsis* causes male sterility, which mimics the phenotype observed in rice flowers infected with the virus. It will be interesting to examine whether RBSDV P7-1, like RDV Pns10, can function as a viral suppressor of RNA silencing.

In this study, we determined that the male sterility of P7-1-OE transgenic plants was due to the failure of anther dehiscence and pollen release, which may have been caused by the lack of lignification in the anther endothecium (Figure 4 and 5). Actually, secondary lignified thickening in the endothecium is critical for providing the mechanical force required for anther dehiscence [22], [27], [28]. This mechanism has been demonstrated experimentally by analyzing *Arabidopsis* mutants such as *myb26* [29]–[31], *NST1* (*SECONDARYWALL THICKENING PROMOTING FACTOR1*), and *NST2* [32]. In these mutants, reduced lignification of anther endothecium results in anther indehiscent and male sterility. Furthermore, the triple *ccc* mutant, which carries mutations in monolignol biosynthesis genes, including *CCR1* (*cinnamoyl CoA reductase1*), *CAD c* (*cinnamyl alcohol dehydrogenase c*), and *CAD d*, displays severe male sterility due to the lack of lignification in the anther endothecium [19]. Here, we showed that the transcriptional levels of the lignin biosynthesis genes *4CL*, *CCoAOMT*, and *C3H* were reduced in the flowers of RBSDV P7-1 transgenic plants compared with the flowers of wild-type plants (Figure 5), although the expression levels of *CCR1*, *CAD c*, and *CAD d* were unchanged in the transgenic plants (data not shown). Thus, the reduced lignification of anthers is responsible for male sterility in P7-1-OE transgenic plants.

It is well-established that secondary lignification in cell walls is dependent on the oxidative coupling of monolignol to form the polymer lignin, which is catalyzed by H_2_O_2_-dependent peroxidases and O_2_-dependent laccase [33], [34]. In the endodermal cells of roots, subcellular precision of lignin polymerization is achieved by restricted, localized peroxidase activity and the production of ROS substrate [35]. In the secondary thickening that occurs in anthers, lignin polymerization is dependent on the H_2_O_2_ contents. For example, *Arabidopsis* plants ectopically expressing *gamma carbonic anhydrase 2* (*CA2*) have significantly lower ROS contents than wild type, which results in a reduction in the H_2_O_2_-dependent polymerization pathway for lignin formation during anther development and accordingly, causes male sterility [36]. Our data also show that ectopic expression of RBSDV P7-1 in *Arabidopsis* causes male sterility in conjunction with low ROS contents in anthers compared with wild-type plants (Figure 6). Thus, it is reasonable to speculate that the male sterility of P7-1 transgenic plants is primarily achieved by the combinatorial action of reduced monolignol and ROS contents. In the future, it will be important to untangle the relationship between reduced production of ROS or lignin and RBSDV tubular protein P7-1. Such information would significantly deepen our mechanistic understanding of the role of RBSDV P7-1 in viral virulence.

## Supporting Information

Figure S1 **Molecular identity of RBSDV P7-1 transgenic** ***Arabidopsis*** **plants.** (A) Selection of *P7-1* transgenic lines on MS medium with kanamycin or without kanamycin (MS). (B) Genome DNA PCR showing that *P7-1* was integrated as a unit in the transgenic lines. *EF1α* was used to control for DNA loading. (C) qRT-PCR analysis showing the expression of *P7-1* in wild-type and three independent transgenic lines using the *EF1a* gene as an internal standard. Results were presented as means±SE from three replications.
(TIF)

Figure S2 **Alexander staining of pollen of** ***P7-1*** **transgenic and wild-type** ***Arabidopsis*** **plants.** (A) Cytological comparisons of P7-1 transgenic lines (P7-1-OE) and wild type (WT) *Arabidopsis* anthers during development. (B, C) Alexander staining pollen of P7-1 transgenic (P7-1-OE) and wild-type (WT) *Arabidopsis*.
(TIF)

Figure S3 **Hand-pollinated pollen grains from P7-1 transgenic plants could fertilize egg cells.**
(TIF)

Figure S4 **NO detected by staining with DAF-FM DA in P7-1 transgenic (P7-1-OE) and wild-type (WT)** ***Arabidopsis*** **anthers.**
(TIF)

Figure S5 **O_2_^−^ detected by staining with NBT in P7-1 transgenic (P7-1-OE) and wild-type (WT)** ***Arabidopsis*** **anthers.**
(TIF)

Table S1 **List of primers used in this study.**
(DOC)
