# Supplementary figures and images for: Overexpression of Rice Black-Streaked Dwarf Virus P7-1 in Arabidopsis Results in Male Sterility Due to Non-Dehiscent Anthers
Source: PLoS One. 2013 Nov 15;8(11):e79514. doi: 10.1371/journal.pone.0079514 (PMC3829848; doi:10.1371/journal.pone.0079514)

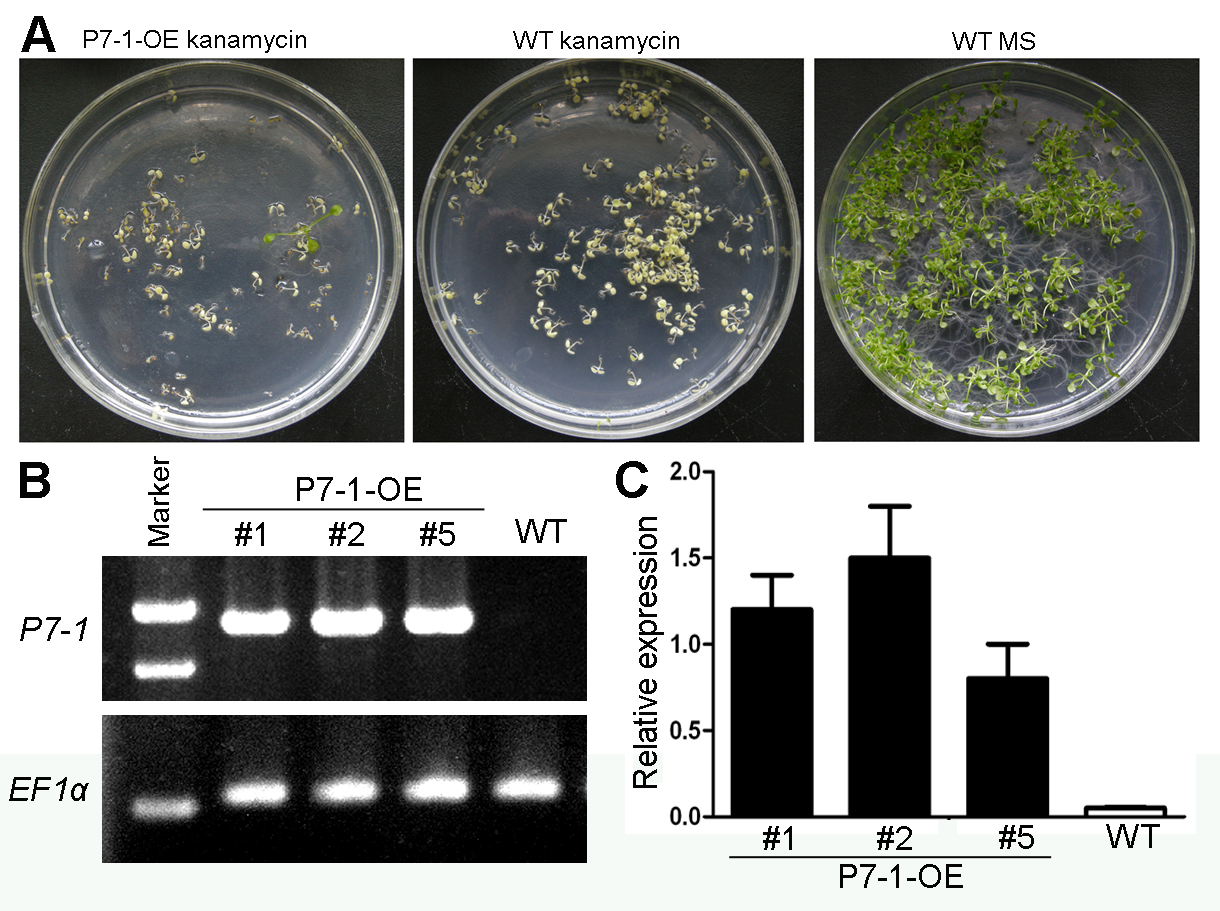

Supplement: Figure S1 — Molecular identity of RBSDV P7-1 transgenic Arabidopsis plants. (A) Selection of P7-1 transgenic lines on MS medium with kanamycin or without kanamycin (MS). (B) Genome DNA PCR showing that P7-1 was integrated as a unit in the transgenic lines. EF1α was used to control for DNA loading. (C) qRT-PCR analysis showing the expression of P7-1 in wild-type and three independent transgenic lines using the EF1a gene as an internal standard. Results were presented as means±SE from three replications. (TIF) [file pone.0079514.s001.tif]

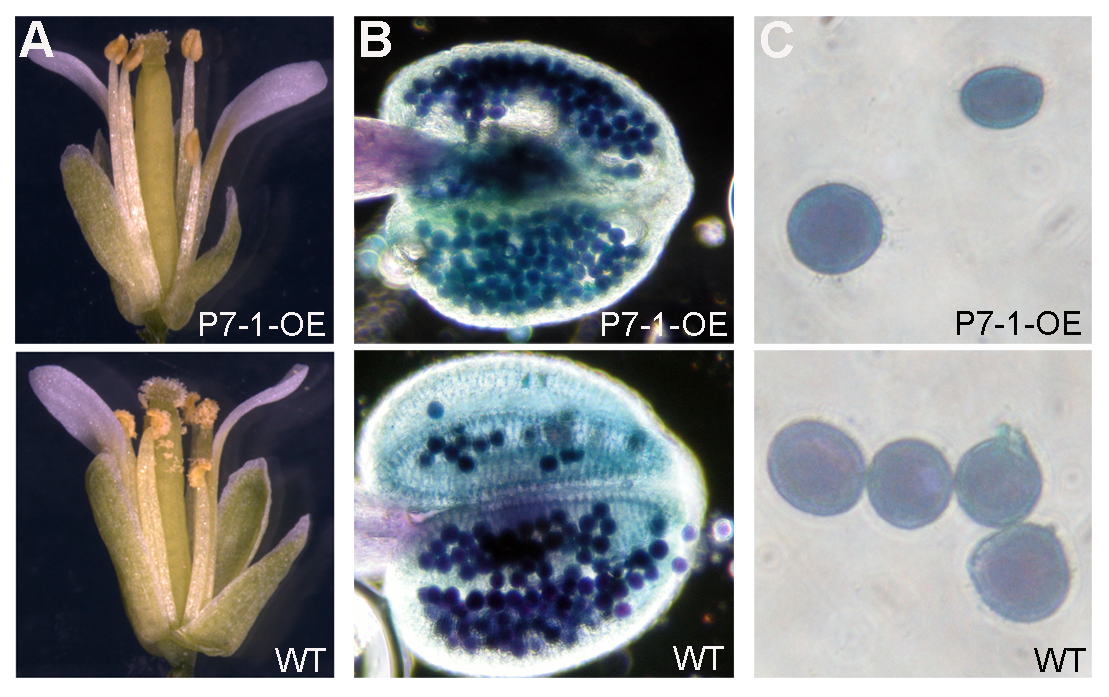

Supplement: Figure S2 — Alexander staining of pollen of P7-1 transgenic and wild-type Arabidopsis plants. (A) Cytological comparisons of P7-1 transgenic lines (P7-1-OE) and wild type (WT) Arabidopsis anthers during development. (B, C) Alexander staining pollen of P7-1 transgenic (P7-1-OE) and wild-type (WT) Arabidopsis. (TIF) [file pone.0079514.s002.tif]

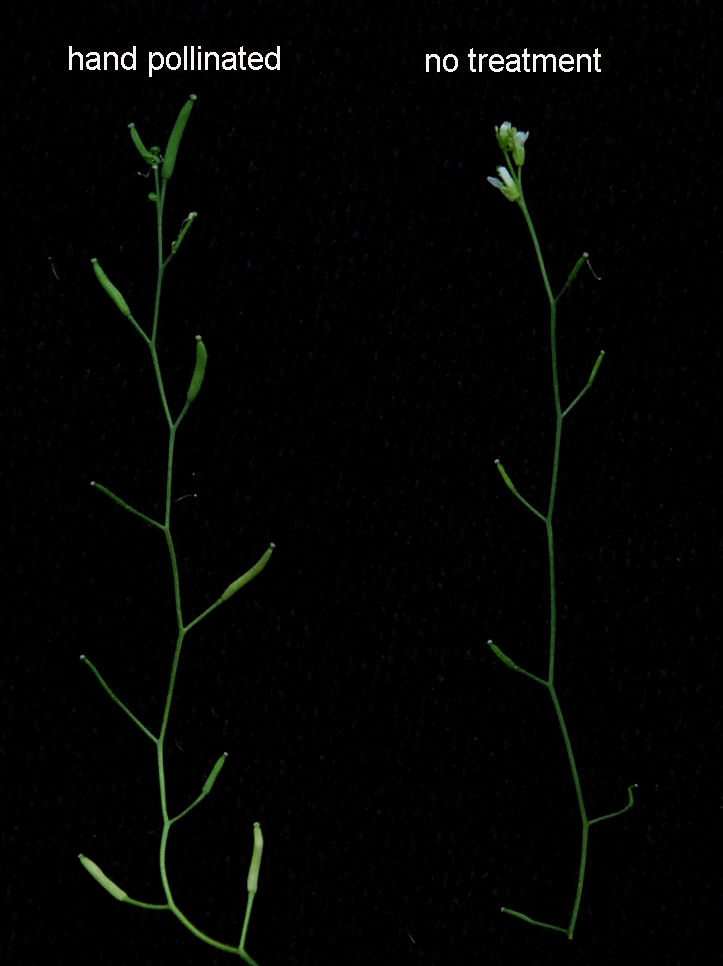

Supplement: Figure S3 — Hand-pollinated pollen grains from P7-1 transgenic plants could fertilize egg cells. (TIF) [file pone.0079514.s003.tif]

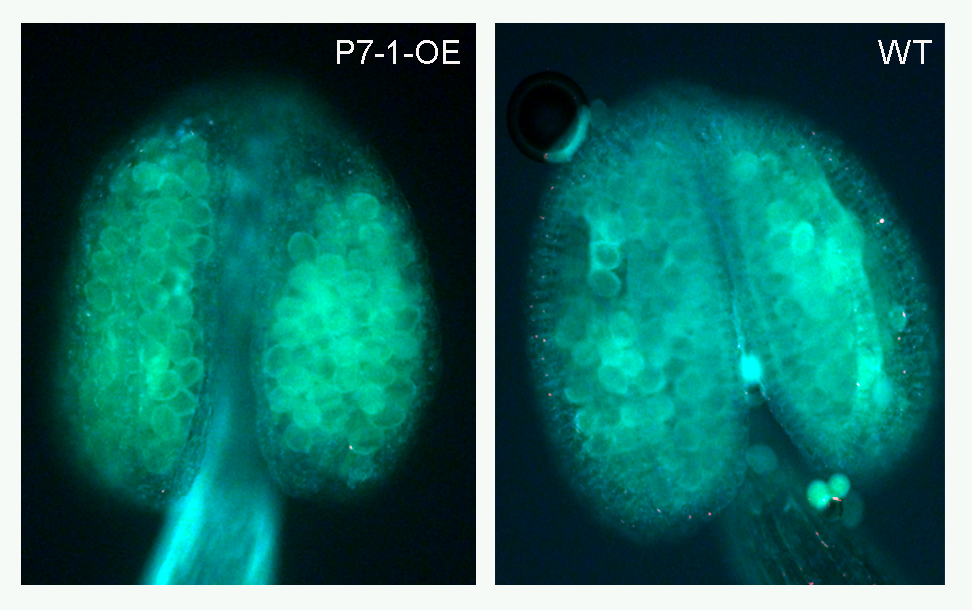

Supplement: Figure S4 — NO detected by staining with DAF-FM DA in P7-1 transgenic (P7-1-OE) and wild-type (WT) Arabidopsis anthers. (TIF) [file pone.0079514.s004.tif]

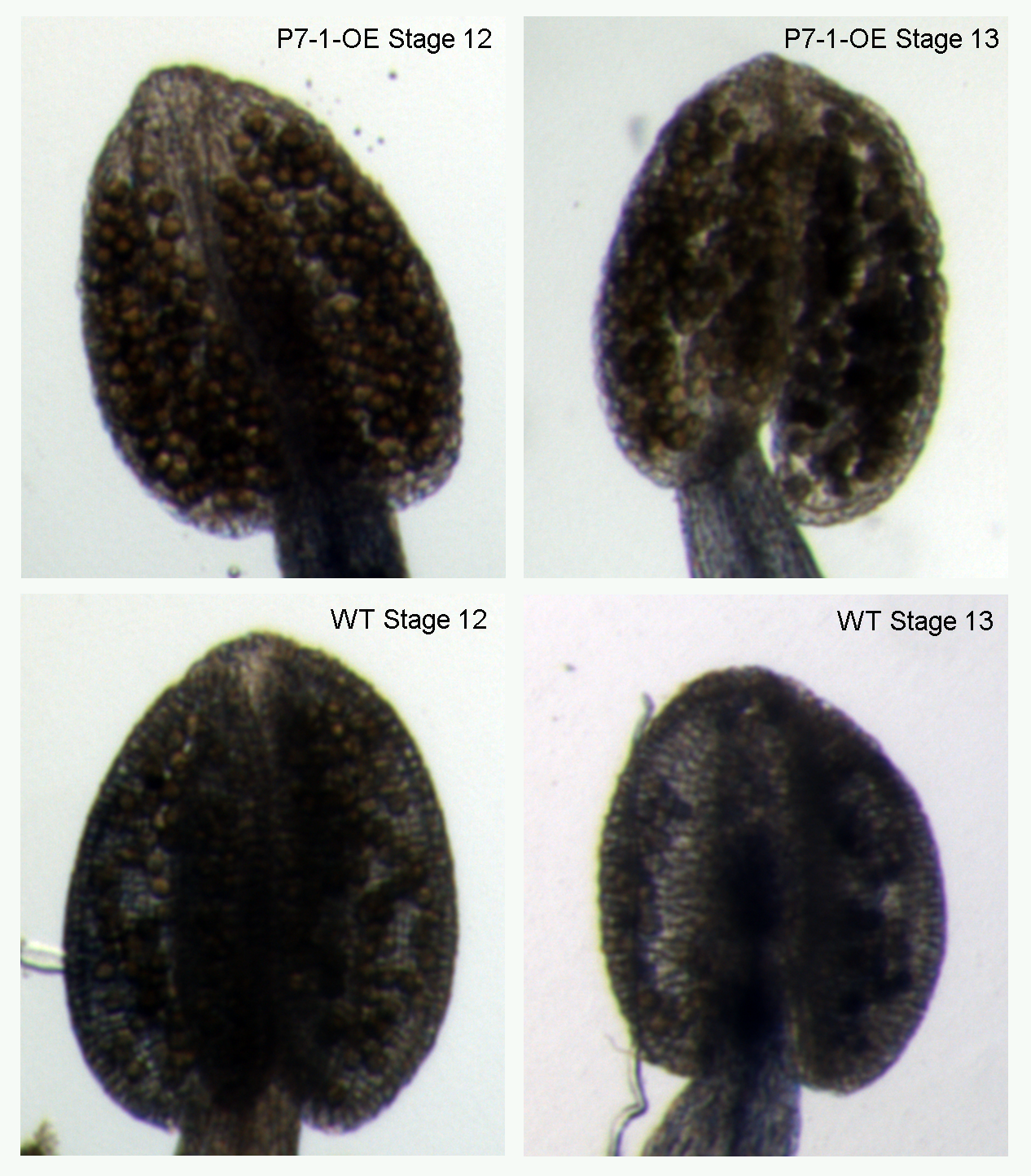

Supplement: Figure S5 — O2− detected by staining with NBT in P7-1 transgenic (P7-1-OE) and wild-type (WT) Arabidopsis anthers. (TIF) [file pone.0079514.s005.tif]
