# Supplementary material for: Overexpression of Rice Black-Streaked Dwarf Virus P7-1 in Arabidopsis Results in Male Sterility Due to Non-Dehiscent Anthers
Source: PLoS One. 2013 Nov 15;8(11):e79514. doi: 10.1371/journal.pone.0079514 (PMC3829848; doi:10.1371/journal.pone.0079514)
Supplement: Table S1 — List of primers used in this study. (DOC) [file pone.0079514.s006.doc]

**Table S1.** List of primers used in this study.

| Gene name | Primers sequence | Product size (bp) | Purpose |
| --- | --- | --- | --- |
| *RBSDVP7-1* | 5’- CG*GAGCTC*ATGGATAGACCTGCTCG -3’  5’- CG*GGATCC*TTAAGCAGAAGGAGATGA -3’ | 1089 | Transgenic and genome PCR |
| *EF1α* | 5’- GGCTGCTGAGATGAACAA -3’  5’- GTGGTGGAGTCAATGATAAG -3’ | 225 | qRT-PCR and  genome PCR |
| *RBSDVP7-1* | 5’- CTCTTGGCAACGCTTTCATA -3’  5’- ATGTTTGTTGGGCGGCTACT -3’ | 168 | qRT-PCR |
| *4CL1* | 5’- AGAACCCGAATCTTTATTTCCAC -3’  5’- CTCAAATCATACTTCTCCGTCTCC -3’ | 250 | qRT-PCR |
| *C3H* | 5’- CGTGGTTTCTAATAGCGGTGGCG -3’  5’- CGGCGAGTTTCTGGTCGTGTTCT -3’ | 273 | qRT-PCR |
| *CCoAMT* | 5’- GACAAATCAAGCTACGTCAACTTCC -3’  5’- TCCTCCGCCACAAAACCAAACCACA -3’ | 107 | qRT-PCR |
| *cAPX* | 5’- ACTCTGGGACGATGCCACAAG -3’  5’- TCTCGACCAAAGGACGGAAAA -3’ | 180 | qRT-PCR |
| *APX2* | 5’- GGTCGGATGGGACTCAAT -3’  5’- AGAGCCTTGTCGGTTGGT -3’ | 194 | qRT-PCR |
| *FSD1* | 5’- ACCAGGTGGTGGAGGAAA -3’  5’- AAGCAATGGGAAAGAGCC -3’ | 214 | qRT-PCR |
| *RBOHA* | 5’- GGAAGAGAGTGTGGGTGATGGC -3’  5’- TAACTTGGTTTTGGTCCGGAGC -3’ | 218 | qRT-PCR |
| *RBOHB* | 5’- GCGAGACGAAACCATCAACACA -3’  5’- CCTCATCCCAGCCTCTACCAAC -3’ | 200 | qRT-PCR |
